# Genetic Analysis of Rare Disorders: Bayesian Estimation of Twin Concordance Rates

**DOI:** 10.1007/s10519-012-9547-9

**Published:** 2012-06-19

**Authors:** Stéphanie M. van den Berg, Jacob vB. Hjelmborg

**Affiliations:** 1 Department of Research Methodology, Measurement and Data Analysis, University of Twente, P.O. Box 217, 7500 AE Enschede, The Netherlands; 2 Department of Biostatistics, University of Southern Denmark, Odense, Denmark; 3 The Danish Twin Registry, University of Southern Denmark, Odense, Denmark

**Keywords:** Methodology, Prior information, Rheumatoid arthritis, Cleft lip

## Abstract

Twin concordance rates provide insight into the possibility of a genetic background for a disease. These concordance rates are usually estimated within a frequentistic framework. Here we take a Bayesian approach. For rare diseases, estimation methods based on asymptotic theory cannot be applied due to very low cell probabilities. Moreover, a Bayesian approach allows a straightforward incorporation of prior information on disease prevalence coming from non-twin studies that is often available. An MCMC estimation procedure is tested using simulation and contrasted with frequentistic analyses. The Bayesian method is able to include prior information on both concordance rates and prevalence rates at the same time and is illustrated using twin data on cleft lip and rheumatoid arthritis.

**Electronic supplementary material:**

The online version of this article (doi:10.1007/s10519-012-9547-9) contains supplementary material, which is available to authorized users.

## Introduction

Methods for the analysis of categorical data from twins have been widely studied (Bartfay et al. 1999; Betensky et al. 2001; Donner et al. 1995; McGue 1992; Ramakrishnan et al. 1992; Shoukri et al. 2003; Smit 1974; Witte et al. 1999 among others) with many applications. There are several measures of association, each having different properties. The case-wise concordance rate is useful in many settings and is easy to interpret. It is defined as the conditional probability of being affected, given that a family member is affected. The family member is often a sibling. In the analysis of dichotomous variables measured in twins, it is useful to estimate case-wise concordance rates separately for monozygotic (MZ) and dizygotic (DZ) twin pairs. If twin concordance rates exceed the prevalence rate, this is an indication that familial factors play a role. These familial factors can be of genetic or environmental origin (or both). If in addition the concordance rate in MZ twins exceeds the one in DZ twins, this suggests that the familial clustering has, at least in part, a genetic origin. Such an analysis of concordance rates in twins is often used before applying the variance component models of quantitative genetics with probit link functions, known as biometric liability or threshold models (Sham 1998). A link to quantitative genetics via the multilocus model for the case-wise concordance to the prevalence is given in Risch (1990). The advantage of analysing concordance rates over the application of threshold models is that it does not involve any strong assumptions such as a normally distributed continuous latent trait.

Here we develop a Bayesian approach to model twin data with dichotomous outcomes, estimate case-wise concordance rates and test for the presence of a heritable component. Inference on prevalence and concordance rates can be based on Maximum Likelihood (ML) principles and asymptotic expressions of their standard deviations can be derived (Witte et al. 1999). The ML method works well with large sample sizes and high prevalence and concordance rates, but not when prevalence and concordance rates are low. ML point estimates may be correct but the confidence intervals are mainly incorrect when the true values are near the boundary of the parameter space (i.e., near 0 or 1). In those cases, the likelihood function no longer approximates a normal distribution, especially in the case of relatively small sample size.

A Bayesian approach with Markov-chain Monte Carlo (MCMC) sampling provides information about the shape of the posterior distribution. In the case of non-informative priors, the posterior distribution is proportional to the likelihood function, and therefore allows more accurate inference. Additionally, a Bayesian approach can take into account prior information on disease prevalence and concordance rates coming from twin and non-twin studies that are often available, which may help increase statistical power. For an introduction to the core concepts of Bayesian data analysis and MCMC estimation, see Gelman et al. (2004).

In the “Method” section a parametrization is presented and an MCMC sampling scheme for estimation is chosen. In the “Simulation studies” section the method is tested in two simulation scenarios and in the “Application to cleft lip” section we apply the method to twin data on cleft lip, both with and without prior information on prevalence. The “Other scenarios with prior information” section discusses more elaborate scenarios where there is both prior information on prevalence and concordance rates. This is illustrated using data sets on rheumatoid arthritis.

## Method

### Concordance rates: setting and notation

Suppose we have health data collected from twin pairs in a population-based sample, and we know each individual’s status: affected or healthy. One could tabulate such data from twins in a 2 × 2 crosstable. Under the often reasonable assumption that twins within a pair are exchangeable, one could simplify the tabulation by using a 3 × 1 vector **y** = {*y*_11_, *y*_*d*_, *y*_00_}′, counting the number of twin pairs where both are affected as *y*_11_, the number of discordant twin pairs as *y*_*d*_ (i.e., *y*_*d*_ = *y*_10_ + *y*_01_ for counts *y*_10_ and *y*_01_ of discordants) and the number of healthy twin pairs as *y*_00_. The likelihood of the data can then be described using a multinomial distribution with probability parameters *p*_11_, *p*_*d*_ and *p*_00_, respectively.

These probabilities in turn can be conceived of as functions of the prevalence of the disease and the degree of dependence within twin pairs. There are many different ways of parametrizing the probabilities. One could choose to use a prevalence parameter π and a concordance rate *q*, where *q* is the conditional probability of being affected, given that the co-twin is affected. However, in a Bayesian model, this parametrization is not invariant with regards to the labeling of affected/unaffected. Setting up particular informative priors for π and *q* would lead to different models if labels were switched. Since we want to generalize the model to traits that are not clearly directional, (e.g., curly or straight hair), we prefer a parametrization that is independent of labeling. Moreover, since the objective of the twin studies is making inference about independence or lack thereof in 2 × 2 tables, the prior on model parameters should not be biased with regards to independence. With a uniform prior on *q*, but an informative prior on π, the expected difference between these two parameters will not be zero, which implies dependence. Of course we need a parametrization that avoids such an implicit prior probability of dependency.

We therefore parametrize the model in terms of prevalence π and δ, where δ is the difference between the probability of being affected conditional on the co-twin being affected, and the probability of being affected conditional on the co-twin not being affected, that is, the Kendall-type measure expressed by

1 $$ \delta = P(\hbox{twin affected}|\hbox{co-twin affected}) - P(\hbox{twin affected}|\hbox{co-twin not affected}) $$

With some algebra we get the expression for the concordance rate, *q*,

2 $$ q = P(\hbox{twin affected}|\hbox{co-twin affected})=\delta(1-\pi)+\pi $$

The multinomial probability parameters can then be described as

3 $$ \begin{aligned} p_{11}&= \pi q = \delta\pi(1-\pi)+ \pi^2\\ p_d&= p_{10}+p_{01} = 2\pi (1-q) = 2\pi(1-\delta(1-\pi)-\pi) \\ p_{00}&=1-p_{11}-p_d=1+\pi(q-2)= 1+ \pi(\delta(1-\pi)+\pi-2). \end{aligned} $$

To avoid negative values for the multinomial probabilities, however, one needs the constraint $q > 2 - \frac{1}{\pi}$ and therefore

4 $$ \delta>\frac{\pi-1}{\pi} $$

Dependence is indicated when δ is clearly different from 0. If δ > 0 this indicates that there is positive familial resemblance. If δ for MZ twins is greater than δ for DZ twins, that is, if δ^MZ^ > δ^DZ^, this suggests a genetic origin for at least some of this familial resemblance.

Alternatively one can focus on the concordance rates that are a function of π and the δ’s. To determine whether familial clustering of a disease in sib pairs is at least partly genetically mediated, it is necessary to show that the concordance rate observed in MZ twin pairs is higher than the concordance rate observed in DZ twin pairs, in other words, that *q* ^MZ^ > *q* ^DZ^. But for the reasons alluded to above, we parametrize the model in terms of δ rather than *q*. By transforming δ and π back to *q*, using Eq. 2, we can still make inference on concordance rates. Such back-transformation of parameters is straightforward in a sampling approach such as the one applied here.

For the Bayesian model we assume exchangeability of twins within pairs (i.e., no effects of being first-born), and identical prevalence in MZ twins, DZ twins, and singletons. We also assume that the numbers of observed MZ and DZ twin pairs are fixed. We assume independence parameter δ and prevalence parameter π a priori independent, save for a constraint that ensures positive expected cell probabilities. Alternatively, based on prior knowledge one might prefer dependent priors for the δs. For example, one could observe that usually in twin studies, for most traits, when we see dependence in MZ twins, we also see dependence in DZ twins. This could be modeled along the lines of a Howard prior (Howard 1998). However, since it is not straightforward how to quantify that observation across traits into a correlation between dependence parameters δ^MZ^ and δ^DZ^, we prefer to assume independence and let only the available data about the trait in question inform us about their values.

For the likelihood function, the only parameters of importance are prevalence π and dependence parameters δ^MZ^ and δ^DZ^. Let **y** ^MZ^ and **y** ^DZ^ denote the 3 × 1 data vectors for the MZ and DZ twin pairs, respectively. The joint distribution of model parameters and data can be factorized as

$$ p(\pi, \delta^{\rm MZ}, \delta^{\rm DZ}, {\bf y}^{\rm MZ}, {\bf y}^{\rm DZ})=p(\pi,\delta^{\rm MZ},\delta^{\rm DZ})p({\bf y}^{\rm MZ}|\pi, \delta^{\rm MZ})p({\bf y}^{\rm DZ}|\pi, \delta^{\rm DZ}), $$

so that the likelihood is proportional to the product of two multinomials:

$$ \begin{aligned} L(\pi, \delta^{\rm MZ}, \delta^{\rm DZ}| {\bf y}^{\rm MZ}, {\bf y}^{\rm DZ}) & \propto \left(p_{11}^{\rm MZ}\right)^{y_{11}^{\rm MZ}} \left(p_{d}^{\rm MZ}\right)^{y_{d}^{\rm MZ}} \left(p_{00}^{\rm MZ}\right)^{y_{00}^{\rm MZ}}\\ & \times \left(p_{11}^{\rm DZ}\right)^{y_{11}^{\rm DZ}} \left(p_{d}^{\rm DZ}\right)^{y_{d}^{\rm DZ}} \left(p_{00}^{\rm DZ}\right)^{y_{00}^{\rm DZ}}. \end{aligned} $$

### Bayesian estimation

In Bayesian analysis, the joint posterior distribution for model parameters is proportional to the product of the likelihood function and the joint prior distribution. Here we assume that the degree of dependence is not related to the prevalence, accept for the constraint in Eq. 4. In addition, as indicated above, we assume the dependence parameters for MZ and DZ twins to be independent. We therefore factorize the joint prior as

$$ p(\pi, \delta^{\rm MZ}, \delta^{\rm DZ})= p( \pi)p(\delta^{\rm MZ}|\pi)p(\delta^{\rm DZ}|\pi) $$

For parameter π we use a Beta prior,

$$ \pi \sim Beta(\alpha_1,\alpha_2)\quad \alpha_1,\alpha_2 \in{\mathbb{R}}^+ $$

For hyperparameters α_1_ and α_2_ one can choose 1 if there is no prior information on disease prevalence. If prior studies are available, for instance from general population samples, one can use the total number of affected individuals, *n*_1_, and the total number of non-affected individuals, *n*_2_, and add them to the non-informative prior *Beta*(1,1), which results in the informative prior *Beta*(1 + *n*_1_,1 + *n*2). This informative prior is exactly proportional to the likelihood for the prevalence given the data *n*_1_ and *n*_2_ in a binomial model. In other words, the prior distribution reflects all knowledge about prevalence gained from the earlier studies.

For parameters δ^MZ^ and δ^DZ^ we use independent truncated scaled Beta distributions

$$ \begin{aligned} p(\delta^{\rm MZ}|\pi, \beta_1, \beta_2) &\propto \frac{(\delta^{\rm MZ}+1)^{\beta_1-1}(1-\delta^{\rm MZ})^{\beta_2-1}}{2^{\beta_1+\beta_2-1}} I(\delta^{\rm MZ}), \quad \delta^{\rm MZ} \in [-1,1]; \beta_1,\beta_2 \in {\mathbb{R}}^+\\ p(\delta^{\rm DZ}|\pi, \gamma_1, \gamma_2) &\propto \frac{(\delta^{\rm DZ}+1)^{\gamma_1-1}(1-\delta^{\rm DZ})^{\gamma_2-1}}{2^{\gamma_1+\gamma_2-1}} I(\delta^{\rm DZ}),\quad \delta^{\rm DZ} \in [-1,1]; \gamma_1,\gamma_2 \in {\mathbb{R}}^+ \end{aligned} $$

where the indicator function *I* is given by

$$ I(\delta) = \left\{\begin{array}{ll}1 & \hbox{if}\; \delta > \frac{\pi-1}{\pi}\\ 0 & \hbox{otherwise.}\end{array}\right. $$

If there is no prior information on concordance rates in twins, one chooses the value 1 for hyperparameters β_1_, β_2_, γ_1_, and γ_2_. The case where prior information on concordance rates is available from an earlier study is discussed in “Other scenarios with prior information” section.

In order to make inferences regarding the model parameters, we set up an MCMC sampling scheme to sample from the joint posterior distribution. In order to make the MCMC sampling as easy as possible, sampling from normal distributions, we first transform the parameters to the real line by using $\lambda=\hbox{ln}\frac{\pi}{1-\pi}\; \hbox{and}\;\mu= \hbox{ln}\frac{\delta+1}{ 1-\delta}.$ The joint posterior distribution of parameters λ,  μ^MZ^ and μ^DZ^ is then proportional to (note the Jacobian term due to the transformation)

5 $$ \begin{aligned} p(\lambda,\mu^{\rm MZ},\mu^{\rm DZ}|{\bf y}^{\rm MZ}, {\bf y}^{\rm DZ}) &\propto \left(\frac{\hbox{exp}(\lambda)}{1+\hbox{exp}(\lambda)}\right)^{\alpha_1-1} \left(1-\frac{\hbox{exp}(\lambda)}{1+\hbox{exp}(\lambda)}\right)^{\alpha_2-1}\\ &\times \left(\frac{ \hbox{exp}\left(\mu^{\rm MZ}\right)-1 }{ 1+\hbox{exp}\left(\mu^{\rm MZ}\right)}+1\right)^{\beta_1-1}\left(1-\frac{ \hbox{exp}\left(\mu^{\rm MZ}\right)-1 }{ 1+\hbox{exp}\left(\mu^{\rm MZ}\right)}\right)^{\beta_2-1}\\ &\times \left(\frac{ \hbox{exp}\left(\mu^{\rm DZ}\right)-1 }{ 1+\hbox{exp}\left(\mu^{\rm DZ}\right)}+1\right)^{\gamma_1-1}\left(1-\frac{ \hbox{exp}\left(\mu^{\rm DZ}\right)-1 }{ 1+\hbox{exp}\left(\mu^{\rm DZ}\right)}\right)^{\gamma_2-1} \\ &\times\frac{\hbox{exp}(\lambda)}{(1+\hbox{exp}(\lambda))^2} \frac{\hbox{exp}(\mu^{\rm MZ})}{(1+\hbox{exp}(\mu^{\rm MZ}))^2} \frac{\hbox{exp}(\mu^{\rm DZ})}{(1+\hbox{exp}(\mu^{\rm DZ}))^2} \\ &\times \left(p_{11}^{\rm MZ}\right)^{y_{11}^{\rm MZ}} \left(p_{d}^{\rm MZ}\right)^{y_{d}^{\rm MZ}} \left(p_{00}^{\rm MZ}\right)^{y_{00}^{\rm MZ}} \left(p_{11}^{\rm DZ}\right)^{y_{11}^{\rm DZ}} \left(p_{d}^{\rm DZ}\right)^{y_{d}^{\rm DZ}} \left(p_{00}^{\rm DZ}\right)^{y_{00}^{\rm DZ}} \end{aligned} $$

with constraint $$\hbox{max}(-1, \frac{\pi-1}{\pi}) < \frac{ \hbox{exp}\left(\mu\right)-1 }{ 1+\hbox{exp}\left(\mu\right)} <1.$$

One can sample from this distribution using a Metropolis–Hastings (MH) algorithm (Gelman et al. 2004). Because λ and the two μ parameters have support on the real line, we can use a multivariate Normal distribution as proposal distribution. This can be done in R (R Development Core Team 2005), by writing out a function for the log-transformed joint posterior distribution for λ and μ (without the constraint). The proposal distribution is also not truncated so that a set of parameter values θ = (λ,  μ^MZ^,  μ^DZ^) at iteration *t* that does not satisfy the contraint, leads to θ_*t*_ = θ_*t*−1_.

In a random-walk MH algorithm (Robert et al. 2004) we used a multivariate normal proposal distribution with expectation equal to the parameter values θ_*t*−1_ and covariance matrix equal to the estimated covariance matrix of the posterior based on a Laplace approximation (Tierney 1986). Inference on π and δ can then be based on back-transforming the posterior samples of λ and the μ parameters. Subsequently, inference on concordance rates can be done after backtransforming π and δ parameters. This transformation is applied to all posterior samples of λ and the two μ parameters, using equations $\delta = \frac{ \hbox{exp}\left(\mu\right)-1 }{1+\hbox{exp}\left(\mu\right)} ,\pi= \frac{1}{1+\hbox{exp}(-\lambda)},$ and Eq. 2. As starting values for λ and the two μ parameters, the posterior modes resulting from the Laplace approximation were used. The R script, which makes use of Jim Albert’s LearnBayes package (Albert 2009), is presented in the Appendix.

## Simulation studies

### Independence

The random-walk Metropolis sampling implemented in R (R Development Core Team 2005) was tested with simulation. A data set with data from 100,000 MZ twin pairs and 100,000 DZ twin pairs was simulated using a disease prevalence of 1 % and complete independence, that is, *q* ^MZ^ = *q* ^DZ^ = π = 0.01 (i.e., δ^MZ^ = δ^DZ^ = 0). The simulated data vectors were **y** ^MZ^ = {6, 1876, 98118}′ and **y** ^DZ^ = {12, 2007, 97981}′. Uninformative priors were used, with α_1_ = α_2_ = β_1_ = β_2_ = γ_1_ = γ_2_ = 1 in respective Beta distributions. Simulated posterior values for λ and μ were backtransformed to π, *q* ^MZ^ and *q* ^DZ^. See Supplementary Materials 3 for a plot of the first 100,000 iterations.

**Fig. 1:**
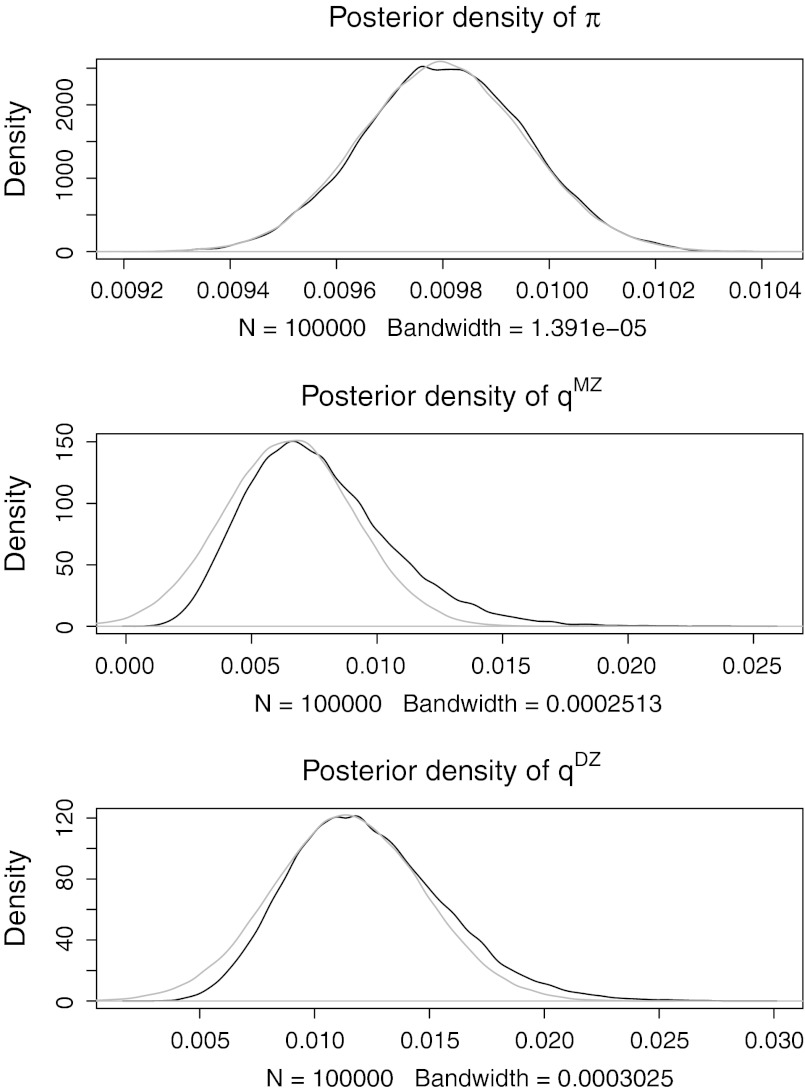
Simulation: independence. Posteriors density plots of π , *q* ^MZ^ and *q* ^DZ^ for a simulated data set. In *gray*, the normal approximation is plotted

Figure 1 shows the marginal posterior densities in black. The 95 % highest posterior density (HPD, Gelman et al. 2004) intervals for π, *q* ^MZ^ and *q* ^DZ^ were (0.95, 1.01 %), (0.27, 1.33 %) and (0.64, 1.94 %), respectively. These are defined as the shortest interval that includes 95 % of the posterior samples and are a Bayesian alternative to frequentist confidence intervals. The HPDs found here all cover the values used in the simulation (i.e., 0.01). In gray, the posteriors are plotted using a normal approximation based on the Laplace method. The normal approximation works well for the prevalence parameter, which can be expected with a data set on 400,000 individual twins. The normal approximation would however give inaccurate intervals for the twin concordance rates, as the posteriors are clearly skewed.

Supplementary Material 4 shows a scatter plot of the three parameters and Supplementary Material 5 shows the autocorrelation in the MCMC chains. MH acceptance rate was 0.45. The slow movement through the posterior can be remedied by using a large number of iterations. Inspecting Supplementary Material 3 and 5 suggests that 100,000 iterations are more than sufficient. This takes about ten seconds with R and a 2.8 GHz processor.

The same dataset was analysed using the software Mx (Neal 2004) for ML-estimation in twin- and family studies. The point estimates for the concordance rates and prevalence were very close to the posterior modes in the Bayesian analysis, but the confidence intervals could not be estimated.

### Familial clustering

A data set with data from 4,000 MZ twin pairs and 6,000 DZ twin pairs was simulated using a disease prevalence of 0.01 and concordance rates of *q* ^MZ^ = 0.40 and *q* ^DZ^ = 0.10. The simulated data vectors were **y** ^MZ^ = {12, 47, 3941}′ and **y** ^DZ^ = {4, 103, 5893}′. Uninformative priors were used, with α_1_ = α_2_ = β_1_ = β_2_ = γ_1_ = γ_2_ = 1. For inference, 100,000 MCMC iterations were run.

**Fig. 2:**
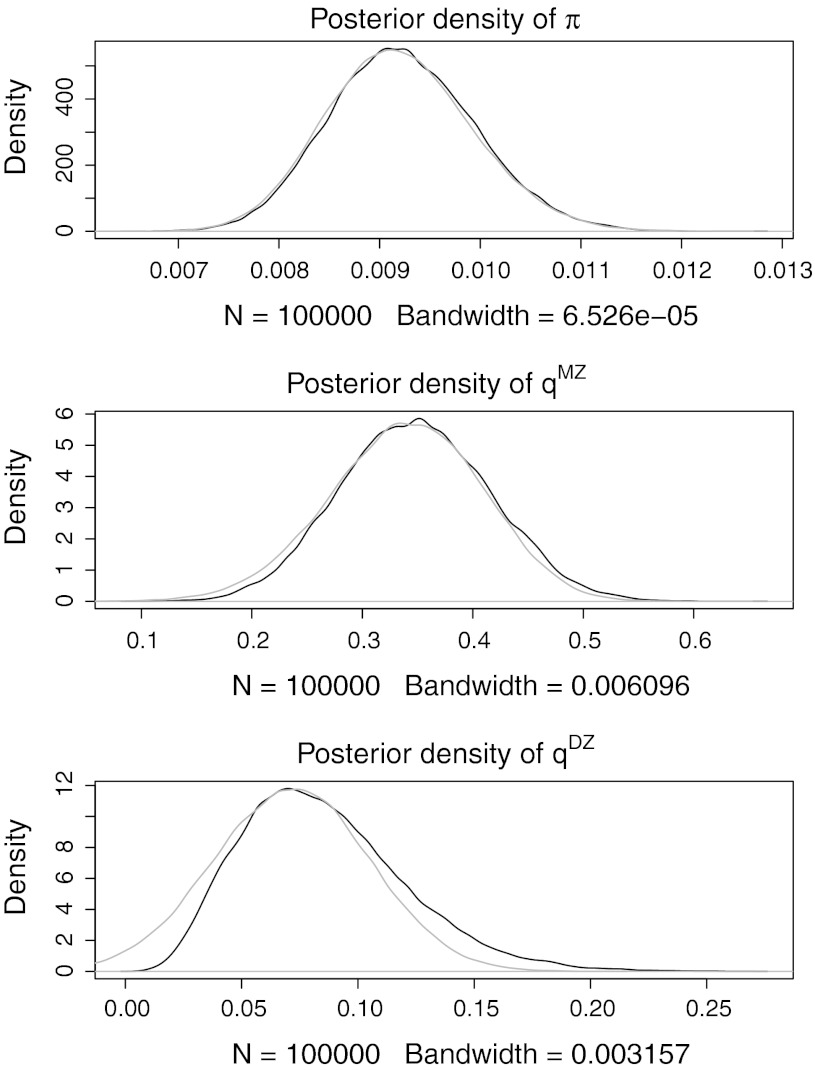
Simulation: familial resemblance. Posteriors density plots of π, *q* ^MZ^ and *q* ^DZ^ for a simulated data set. In *gray*, the normal approximation is plotted

The behavior of the MCMC chain was very similar to the independence scenario in terms of autocorrelation, crosscorrelations and MH acceptance rate. Figure 2 shows the marginal posterior densities. The 95 % highest posterior density intervals for π, *q* ^MZ^ and *q* ^DZ^ were (0.78, 1.07 %), (0.22, 0.48 %) and (0.02, 0.15 %), respectively. The figure also shows that a normal approximation leads to considerably different posterior intervals compared to the MCMC approach, particularly for the relatively small *q* ^DZ^.

The same dataset was analysed using Mx (Neale 2004). The point estimates for the concordance rates and prevalence were again very close to the posterior modes in the Bayesian analysis, but the confidence interval for prevalence could not be estimated. The confidence intervals for the concordance rates were close to the Bayesian HPD intervals.

## Application to cleft lip

Data on cleft lip were analysed coming from Danish boy twins (Grosen et al. 1936). Data vectors were **y** ^MZ^ = {3, 8, 4474}′ and **y** ^DZ^ = {1, 14, 8164}′. Data were first analysed with non-informative priors for all three parameters. Next, based on Statistics Denmark (see Statistics Denmark 2009 and Grosen et al. 2011) we used informative priors for prevalence π. In that data set out of a total of 2,524,359 boys there were 1,693 with cleft lip. For hyperparameters α_1_ and α_2_ we therefore chose 1,694 and 2,522,667, respectively. Both analyses were based on 100,000 MCMC iterations.

Table 1 presents posterior means, medians, SDs, and HPD intervals, both with and without an informative prior on the prevalence. There is clear evidence for familial clustering for cleft lip, given that 0 is not included in the 95 % intervals for the differences between the prevalence and the concordance rates. The difference between the two concordance rates is however not significant, neither with nor without an informative prior. The prior on the prevalence has a clear effect on the estimates for prevalence π: a lower estimate and more precision as indicated by the smaller SD. Additionally, the informative prior has an indirect effect on the estimates of *q* ^MZ^ and *q* ^DZ^: means and medians have clearly shifted. The effect on the SDs illustrates that inclusion of prior information on prevalence affects the statistical power of finding a significant difference between *q* ^MZ^ and *q* ^DZ^.

**Table 1:** Cleft lip in Danish boys: posterior means, posterior SDs, posterior medians, and 95 % highest posterior density (HPD) intervals

	Mean	SD	Median	95 % HPD interval
*Non-informative priors*				
π	0.12 %	0.03 %	0.12 %	(0.08, 0.18 %)
*q* ^MZ^	0.41	0.14	0.40	(0.14, 0.67)
*q* ^DZ^	0.21	0.12	0.20	(0.01, 0.43)
$q^{\rm MZ} - q^{\rm DZ}$	0.20	0.18	0.20	(−0.16, 0.53)
$q^{\rm MZ} - {\pi}$	0.40	0.14	0.40	(0.14, 0.67)
$q^{\rm DZ} - {\pi}$	0.21	0.12	0.19	(0.013, 0.43)
*Informative prior for π*				
π	0.07 %	0.002 %	0.07 %	(0.06, 0.07 %)
$q^{\rm MZ}$	0.36	0.13	0.35	(0.12, 0.62)
$q^{\rm DZ}$	0.16	0.09	0.14	(0.01, 0.34)
$q^{\rm MZ}-q^{\rm DZ}$	0.20	0.16	0.20	(−0.12, 0.51)
$q^{\rm MZ}-{\pi}$	0.36	0.13	0.35	(0.12, 0.62)
$q^{\rm DZ} - {\pi}$	0.16	0.09	0.14	(0.005, 0.34)

The data set without prior information was also analysed using Mx. The point estimate for the prevalence was 0.12 % and equal to the Bayesian posterior mean and median. The point estimates for *q* ^MZ^ was 0.38 and therefore slightly lower than the Bayesian estimates. The point estimates for *q* ^DZ^ was 0.14 and therefore rather different from the Bayesian estimate, which on the basis of a density plot could only partly be explained by the skewness of the posterior (the mode should be smaller than mean and median). The confidence intervals for all three parameters were all similar to the Bayesian HPD intervals.

## Other scenarios with prior information

### Method

The method outlined above showed how prior information on prevalence can be incorporated in the prior density for π. However, it is also possible that there are prior twin studies. These provide not only information on concordance rates but also on prevalence. How to include such information in a new study?

In a situation with no prior information, all values for prior parameters α_1_ etc are set to 1. In such cases with flat priors, the posterior is proportional to the likelihood function. In the case of a prior twin study, the posterior resulting from that prior study with data set **x** is proportional to the likelihood function. When a new study is conducted, the posterior of the prior study should serve as a prior. The posterior of the second study with data set **y** is proportional to the likelihood given **y**, times the prior (being the posterior of the first study). This is in fact proportional to the product of the likelihoods of the two respective studies if we take a flat prior for *p*(π, *q* ^MZ^, *q* ^MZ^),

6 $$ \begin{aligned} p(\pi,\delta^{\rm MZ}, \delta^{\rm MZ}|{\bf x},{\bf y}) &\propto L(\pi, \delta^{\rm MZ}, \delta^{\rm DZ}|{\bf y}) p( \pi, \delta^{\rm MZ}, q^{\rm MZ}|{\bf x})\\ &\propto L(\pi, \delta^{\rm MZ}, \delta^{\rm MZ}|{\bf y} )L(\pi, \delta^{\rm MZ}, \delta^{\rm MZ}|{\bf x} )p(\pi, \delta^{\rm MZ}, \delta^{\rm MZ}) \end{aligned} $$

We can therefore combine the prior information with the new data by analyzing the combined data vectors **z** ^MZ^ = **x** ^MZ^ + **y** ^MZ^ and **z** ^DZ^ = **x** ^DZ^ + **y** ^DZ^ and using the procedure outlined in the “Method” section. Any extra information from studies on prevalence alone can then be included by using an informative prior for π. Below we illustrate this approach by analyzing data on rheumatoid arthritis.

### Application to rheumatoid arthritis

The method of incorporating prior information both from other twin studies and prevalence studies is illustrated using a Danish twin data set on rheumatoid arthritis (The Danish Twin Register 2010; age range: 12-73). The data vectors were **y** ^MZ^ = {4, 58, 7517}′ and **y** ^DZ^ = {2, 126, 11666}′. Analysing this data set using noninformative priors gave results as presented in Table 2. A Finnish twin study (age range: 10+; Aho et al. 1986) found data vectors **x** ^MZ^ = {9, 64, 4064}′ and **x** ^DZ^ = {6, 167, 8983}′. Moreover, a Norwegian study found in a population sample of 356486 (age range: 20-79), a total of 1333 affected people (Kvien et al. 1997). Incorporating such ‘historical data’ on prevalence and concordance rates was accomplished by analysing the summed data vectors **z** ^MZ^ = {13, 122, 11581} and **z** ^DZ^ = {8, 293, 20649} and using π ∼ *Beta*(1334, 355154) with flat scaled Beta priors for δ^MZ^ and δ^DZ^. Note that in this way, each data set is weighted equally. Alternatively, based on the similarity of the data sets (e.g., regarding age ranges), different weights could be used for these other studies, see for example Ibrahim and Chen (2000).

**Table 2:** Rheumatoid arthritis in Danish twins: posterior statistics with and without informative priors

	Mean	SD	Median	95 % HPD interval
*Non-informative priors*				
π	0.52 %	0.04 %	0.51 %	(0.44, 0.59 %)
*q* ^MZ^	0.16	0.06	0.15	(0.05, 0.27)
*q* ^DZ^	0.04	0.02	0.04	(0.01, 0.09)
$q^{\rm MZ} - q^{\rm DZ}$	0.12	0.06	0.11	(−0.002, 0.24)
$q^{\rm MZ} - {\pi}$	0.15	0.06	0.15	(0.04, 0.27)
$q^{\rm DZ} - {\pi}$	0.04	0.02	0.03	(0.0002, 0.08)
*Including prior information*				
π	0.42 %	0.01 %	0.42 %	(0.40, 0.44 %)
$q^{\rm MZ}$	0.15	0.03	0.14	(0.08, 0.22)
$q^{\rm DZ}$	0.04	0.01	0.04	(0.02, 0.07)
$q^{\rm MZ}-q^{\rm DZ}$	0.11	0.04	0.10	(0.04, 0.18)
$q^{\rm MZ}-{\pi}$	0.14	0.03	0.14	(0.08, 0.21)
$q^{\rm DZ}-{\pi}$	0.04	0.01	0.04	(0.01, 0.06)

We used 100,000 iterations with the posterior modes as starting values. As shown by Table 2 the point estimates are slightly affected by the extra information whereas the effects on the posterior SDs and the 95 % HPD intervals are more dramatic. With non-informative priors, the difference between *q* ^MZ^ and *q* ^DZ^ is not significant, whereas with information from other studies included, the evidence for genetic influences on rheumatoid arthritis is clear: with a 97.5 % probability, the difference between *q* ^MZ^ and *q* ^DZ^ is larger than 0.04.

The Danish data set without prior information was also analyzed using Mx. The point estimate for prevalence was equal to the posterior median, but the point estimates for the MZ and DZ concordance rates were both somewhat lower (0.14 and 0.03 respectively) than the Bayesian estimates. The concordance rates confidence intervals were very close to the HPD intervals. For prevalence, the upper bound of the confidence interval could not be estimated.

## Discussion

Here we developed a fully Bayesian approach of estimating case-wise concordance rates. Our method is particularly suited for traits with very low prevalence, where standard methods based on asymptotic theory become unreliable (the normal approximation works only with high information content and/or parameter values far removed from the boundaries of the parameter space). In two simulations studies we showed that particularly for low concordance rates (less than 0.1), the normal approximation of the likelihood function does not hold, as the function is positively skewed (in the case of noninformative priors, the Bayesian posterior distribution has the same shape as the likelihood function).

The data were also analysed using Mx. Mx does not use normal approximation to come up with confidence intervals, but applies a likelihood profile approach. In theory this should result in better estimates for the confidence intervals, but here we observed that, particularly for low values of prevalence and concordance rates, there were computational problems (‘code red’), and failure notices, which made inference unreliable. Moreover, boundary constraints had to be put on the probability parameters, so that they were not too close to 0 and 1. This is of course problematic if the null hypothesis is that the concordance rates are equal to a very low prevalence. One other problem appears to be the constraint of equal prevalences across MZ and DZ twins, since without these constraints no problems were observed.

By using an MCMC algorithm, normal approximations or profile approaches are not necessary as one can directly sample from the posterior distribution. An extra advantage of a Bayesian approach is that it allows a straightforward incorporation of already available knowledge regarding prevalence, or even prior twin studies. In the frequentistic context one can also incorporate such knowledge, but is more tedious. For example, in Mx one could add an extra data group and specify the binomial likelihood for the prevalence parameter given a data set on *n*_1_ affected individuals and *n*_2_ healthy individuals. In contrast, in the Bayesian approach all one has to do is specify the parameter values for the prevalence Beta prior as *n*_1_ + 1 and *n*_2_ + 1, respectively.

Using informative priors increases statistical power. As seen in the “Application to cleft lip” section, even only prior information on prevalence may help to detect a genetic origin of familial clustering. One might feel reluctant to incorporate data from different studies and populations and might note possible differences in genetic background of the populations and in assessment; however, in equal measure one might be reluctant to base an estimate for case-wise concordance solely on two concordant DZ twin pairs, as seen in the Danish arthritis data set. In all situations with low prevalence, estimates are highly sensitive to the number of concordant pairs, where a slight change of two pairs to, for example, one pair has a large impact on point estimates. Therefore, combining studies and increasing total numbers is important in establishing more stable estimates, with accompanying smaller 95 % posterior intervals. If it is felt that some prior studies provide more relevant information than others, a weighting can be applied (see e.g., Ibrahim and Chen 2000). In sum, incorporating other twin and prevalence data may lead to more accuracy and statistical power to detect familial clustering and detecting genetic origins of such clustering.

The presented method is appropriate for research settings with complete ascertainment or where inclusion is not conditional on disease status, for example with population-based twin registries. Nevertheless, the method may be extended to the case of non-complete ascertainment (McGue 1992). The method may also be extended to the multivariate case or equivalently, the case of categorical traits with more than two states. Multinomial log-linear models (see e.g., Forster 2010) can be considered in order to include possible covariates influencing the concordance rates. Further, it is desirable to take time-to-event into account when dealing with possible censorings (e.g., twins that are not yet affected).

By modeling the data using only the three parameters for overall prevalence and MZ and DZ dependencies, the method uses the common assumption that prevalence is equal across zygosity. In cases where prevalence is different across zygosity, for example DZ twinning itself (Hoekstra et al. 2008), the model can be extended to incorporate two different prevalence parameters with separate prior specifications. But the question then arises how to determine whether there are genetic influences: if prevalence is higher in MZ twins than in DZ twins, the expected MZ twin concordance rates assuming complete independence will also be higher than the DZ concordance rate. Or one might have that concordance rates are equal for MZ and DZ twins while the (liability to) the trait is heritable. Hence the scale of which genetic influence is inferred becomes important. Finally, models for genetic heterogeneity as proposed in Risch (1990) in which relative recurrence risks are considered may also be handled from the method proposed in the present paper.

The presented method is novel and has its main merits in its intuitive approach to including prior information and its ability to deal with data sets with very few concordant pairs. Future work will focus on multivariate extensions and the inclusion of covariates such as environmental characteristics (either shared or non-shared), measured genotypes, and time-to-event.

### Electronic supplementary material

Below is the link to the electronic supplementary material.

PDF (1049 KB)

## Appendix: R code

**Table:**

# specify a function logpost() that computes the logposterior distribution (Eq. 5)
logpost<- function(theta, par)
{
ymz <- par$data[,1]; ydz <- par$data[,2];
lambda<- theta[1]; mu.mz<- theta[2]; mu.dz<- theta[3]
alpha1 <- par$alpha1;alpha2<- par$alpha2
beta1 <- par$beta1;beta2<- par$beta2; gamma1 <- par$gamma1; gamma2<- par$gamma2
delta.mz <- (exp(mu.mz)-1)/(1+exp(mu.mz));delta.dz <- (exp(mu.dz)-1)/(1+exp(mu.dz))
pi <- exp(lambda)/(1+exp(lambda))
logl.mz1 <- ymz[1]*(log(pi)+log(delta.mz+ pi*(1-delta.mz)))
logl.mz2 <-ymz[2]*(log(pi)+log(1-delta.mz*(1-pi)-pi))
logl.mz3 <- ymz[3]*log(1+pi*(delta.mz(1-pi)+pi-2))
logl.dz1 <- ydz[1]*(log(pi)+ log(delta.dz + pi*(1-delta.dz)))
logl.dz2 <-ydz[2]*(log(pi)+log(1-delta.dz*(1-pi)-pi))
logl.dz3 <- ydz[3]*log(1+pi*(delta.dz(1-pi)+pi-2))
logpriorlambda <- (alpha1-1)*log(pi) + (alpha2-1)*log(1- pi)
logpriormumz <- (beta1-1)* log((exp(delta.mz)-1)/ (1+exp(delta.mz))+1) + (beta2-1)*log(1- (exp(delta.mz)-1)/ (1+ exp(delta.mz)))
logpriormudz <- (gamma2-1)* log((exp(delta.dz)-1)/ (1+ exp(delta.dz)) +1) + (gamma2-1)*log(1- (exp(delta.dz)-1)/ (1+ exp(delta.dz)))
logJacobian<- lambda-2*log(1+ exp(lambda))+mu.mz-2*log(1+exp(mu.mz))+mu.dz-2*log(1+ exp(mu.dz))
log <-logl.mz1 + logl.mz2 + logl.mz3 + logl.dz1 + logl.dz2 + logl.dz3
log<- log + logpriorlambda + logpriormumz + logpriormudz + logJacobian
return(log)
} # end function logpost

**Table:**

library(LearnBayes) # for functions laplace() and rwmetrop()
# data vectors:
ymz=c (3,8, 4474), ydz= c(1, 14, 8164)
data<- matrix(c(ymz, ydz), 3,2)
# get parameters values for joint posterior:
par <- list(alpha1=1, alpha2=1, beta1=1, beta2=1, gamma1=1,gamma2=1, data=data)
m=100000 # number of MCMC iterations
# laplace approximation of posterior mode and variance:
outlaplace <- laplace(logpost, c(−1,0,0), par)
proposal = list(var=outlaplace$var, scale=1)
out <-rwmetrop(logpost, proposal, start=outlaplace$mode, m, par)
pi <- 1/ (1+ exp(−1*out$par[,1])) # transforming mu into pi
delta.mz <- (exp(out$par[,2])-1) / (1+ exp(out$par[,2]))
delta.dz <- (exp(out$par[,3])-1) / (1+ exp(out$par[,3]))
q <- function(pi, delta){ return(delta*(1-pi)+ pi)}# function that # computes concord rate
qmz <- q(pi,delta.mz) # transforming pi and a delta into a # concordance rate
qdz <- q(pi,delta.dz)
