# Supplementary material for: Genetic Analysis of Rare Disorders: Bayesian Estimation of Twin Concordance Rates
Source: Behav Genet. 2012 Jun 19;42(5):857–65. doi: 10.1007/s10519-012-9547-9 (PMC3442174; doi:10.1007/s10519-012-9547-9)
Supplement: Supplementary file 1 — PDF (1049 KB) [file 10519_2012_9547_MOESM1_ESM.pdf]

## Supplementary Materials

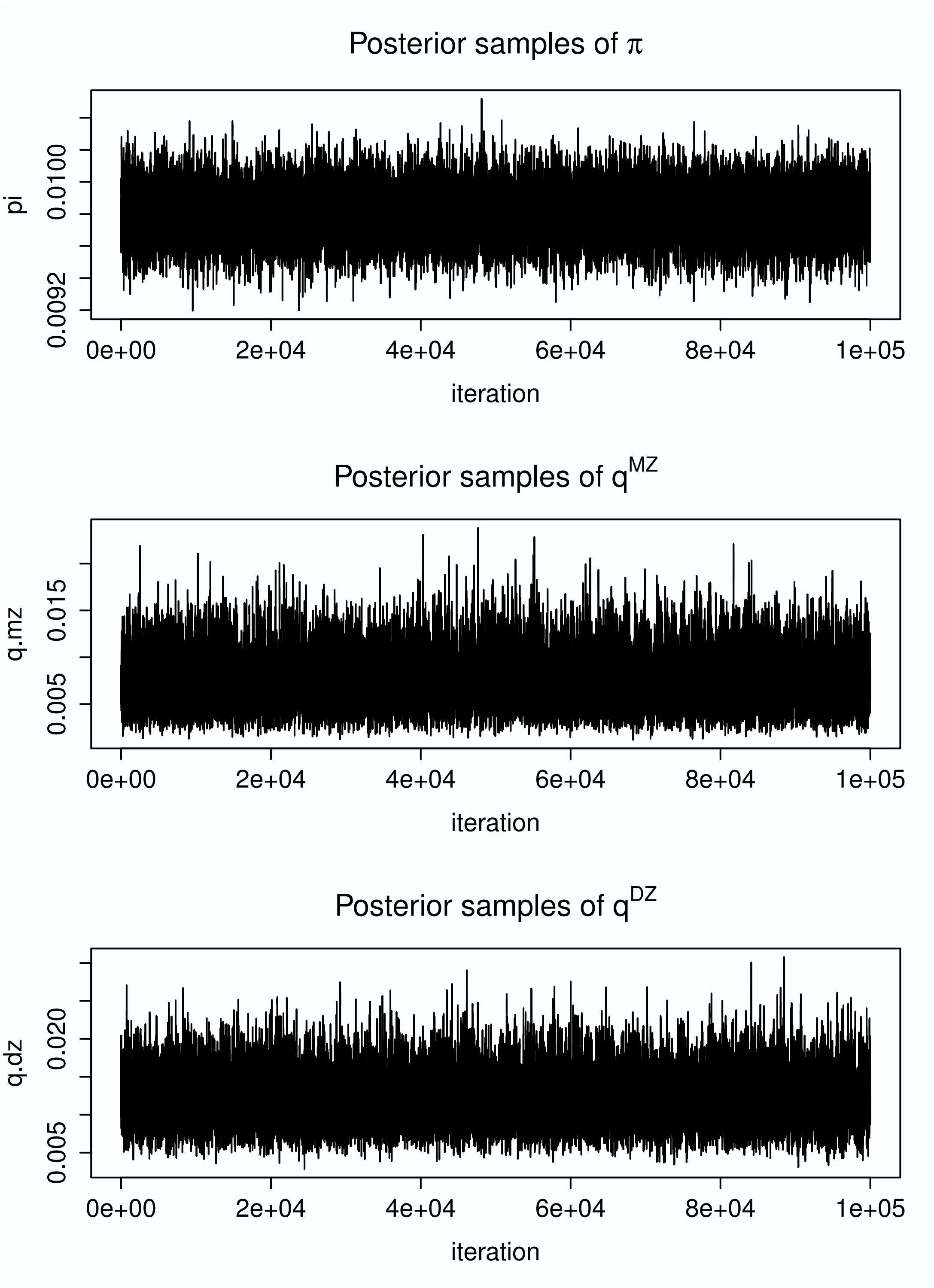


Figure 1: Simulation: independence. Iterations plot for $\pi$, $q^{\mathrm{MZ}}$ and $q^{\mathrm{DZ}}$ for a simulated data set, see section Simulation Studies.

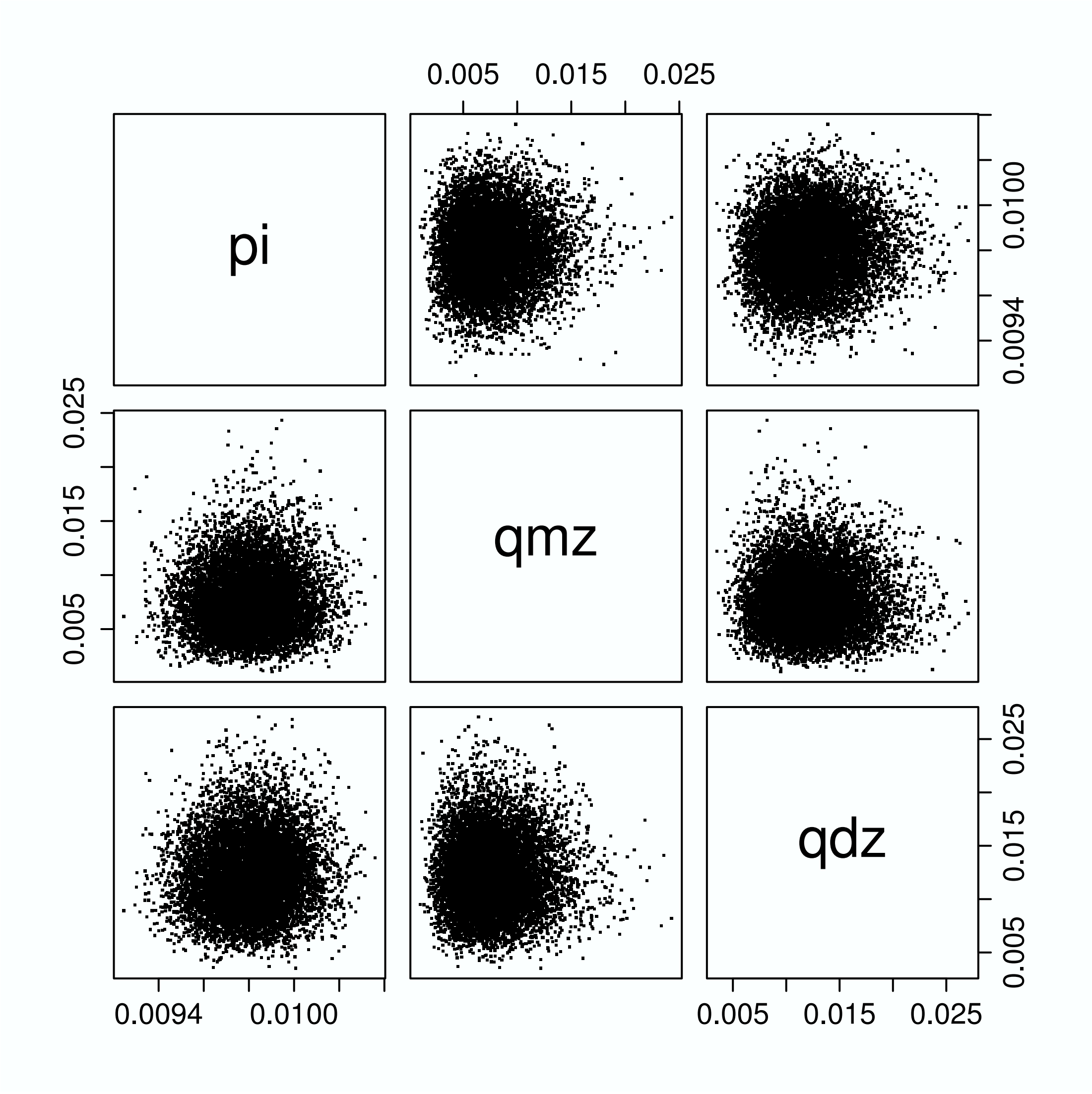


Figure 2: Simulation: independence. Scatter diagram of posterior samples of $\pi$, $q^{\mathrm{MZ}}$ and $q^{\mathrm{DZ}}$ for a simulated data set, see section Simulation Studies.

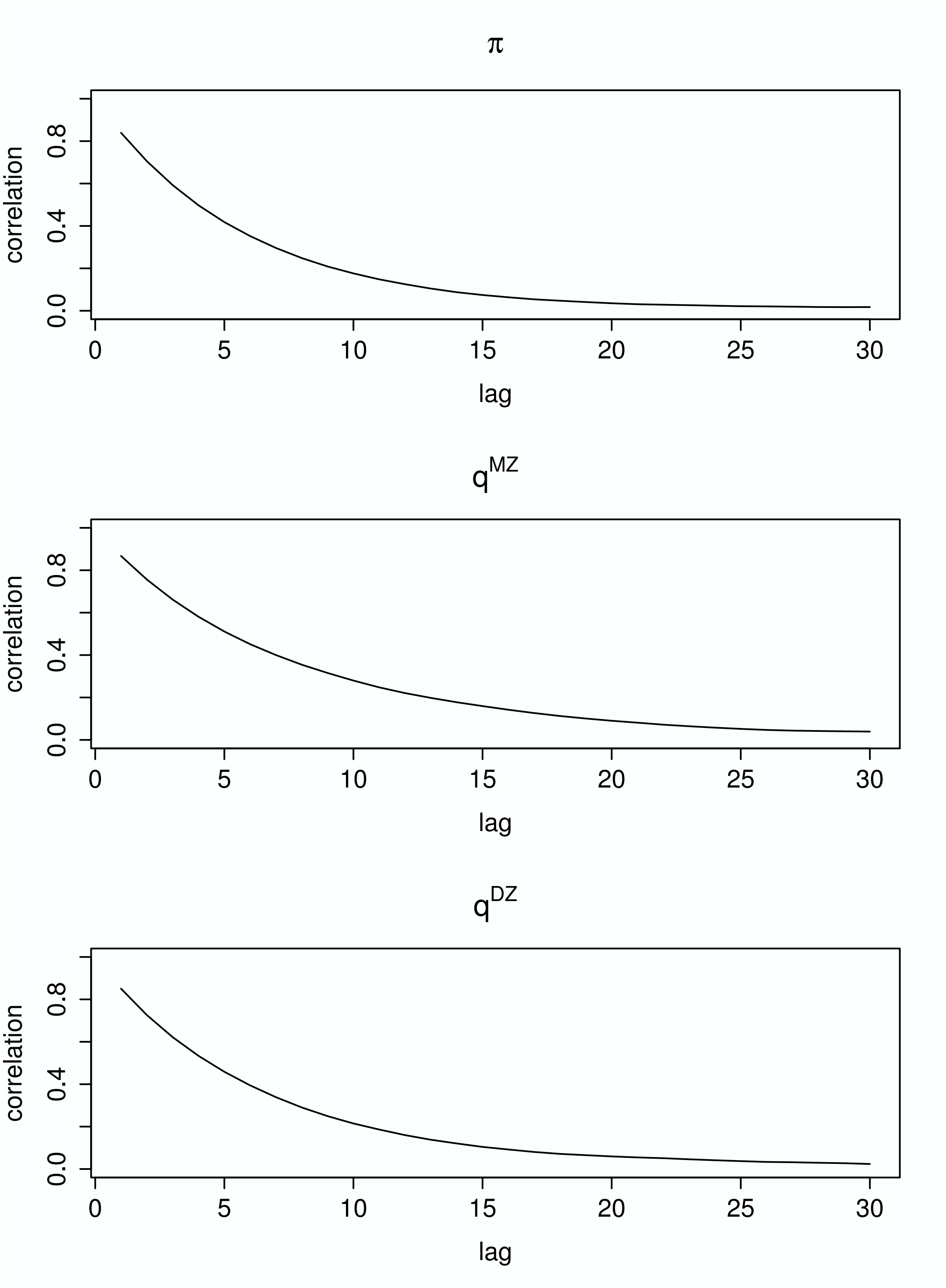


Figure 3: Simulation: independence. Autocorrelation in posterior samples of $\pi$, $q^{\mathrm{MZ}}$ and $q^{\mathrm{DZ}}$ for a simulated data set, see section Simulation studies.
